# Low-serum GTA-446 anti-inflammatory fatty acid levels as a new risk factor for colon cancer

**DOI:** 10.1002/ijc.27673

**Published:** 2012-06-14

**Authors:** Shawn A Ritchie, Jon Tonita, Riaz Alvi, Denis Lehotay, Hoda Elshoni, Su- Myat, James McHattie, Dayan B Goodenowe

**Affiliations:** 1 Phenomenome Discoveries, Inc.Saskatoon, SK, Canada; 2 Department of Population Health, Saskatchewan Cancer AgencyRegina, SK, Canada; 3 Department of Epidemiology, Saskatchewan Cancer AgencySaskatoon, SK, Canada; 4 Saskatchewan Disease Control LaboratoryRegina, SK, Canada; 5 Department of Pathology, University of SaskatchewanSaskatoon, SK, Canada; 6 Division of Gastroenterology, Regina Qu'Appelle Health RegionRegina, SK, Canada

**Keywords:** colorectal cancer, screening, biomarker, inflammation, fatty acid, GTA-446

## Abstract

Gastrointestinal tract acid-446 (GTA-446) is a long-chain polyunsaturated fatty acid present in the serum. A reduction of GTA-446 levels in colorectal cancer (CRC) patients has been reported previously. Our study compared GTA-446 levels in subjects diagnosed with CRC at the time of colonoscopy to the general population. Serum samples and pathology data were collected from 4,923 representative subjects undergoing colonoscopy and from 964 subjects from the general population. Serum GTA-446 levels were determined using a triple-quadrupole tandem mass spectrometry method. A low-serum GTA-446 level was based on the bottom tenth percentile of subjects with low risk based on age (40–49 years old) in the general population. Eighty-six percent of newly diagnosed CRC subjects (87% for stages 0–II and 85% for stages III–IV) showed low-serum GTA-446 levels. A significant increase in the CRC incidence rate with age was observed in subjects with low GTA-446 levels (*p* = 0.019), but not in subjects with normal levels (*p* = 0.86). The relative risk of CRC given a low GTA-446 level was the highest for subjects under age 50 (10.1, 95% confidence interval [C.I.] = 6.4–16.4 in the reference population, and 7.7, 95% C.I. = 4.4–14.1 in the colonoscopy population, both *p* < 0.0001), and declined with age thereafter. The CRC incidence rate in subjects undergoing colonoscopy with low GTA-446 levels was over six times higher than for subjects with normal GTA-446 levels and twice that of subjects with gastrointestinal symptoms. The results show that a low-serum GTA-446 level is a significant risk factor for CRC, and a sensitive predictor of early-stage disease.

Colorectal cancer (CRC) is the second leading cause of cancer death in Canada and it is estimated that 22,200 new diagnoses and 8,900 deaths will occur in 2012.^1^ Financial and societal costs of late-stage diagnosis are significantly greater compared to early-stage diagnosis, owing to increased costs of treatment and poor survival rate.^2^, ^3^ Current screening guidelines are based primarily on fecal occult blood testing and colonoscopy, but these screening approaches continue to struggle with compliance issues,^4^, ^5^ have questionable cost-benefit^6^ and show variable diagnostic performance depending on the method.^7^ New tests that are cost-effective and more acceptable to patients are therefore required to improve CRC screening compliance and early-stage detection rates.

Gastrointestinal (GI) tract acid-446 (GTA-446) is a representative member of a recently identified novel family of circulating long-chain fatty acid metabolites referred to as GTAs, originally discovered by high-resolution metabolomic profiling of serum from patients with CRC and asymptomatic controls.^8^ GTAs, and GTA-446 in particular, have consistently shown reduced levels in CRC patient serum compared to disease-free subjects.^8^, ^9^ The GTA family comprises over 20 members containing between 28 and 36 carbons and ranging in size between 446 and 596 Da. Reduced GTA levels (including GTA-446) in CRC patients are not restored after surgery, chemo or radiation therapy, and are not the result of tumor burden.^9^ The reduction is, therefore, thought to precede tumor formation.

GTA-446 is inversely correlated with age and CRC incidence rate, and exhibits both anti-proliferative and anti-inflammatory activities.^9^, ^10^ The current hypothesis is that a reduction of serum GTA-446 levels over time represents a compromised ability to protect against accumulating chronic inflammation and abnormal cell growth, which ultimately leads to a pro-cancer environment.^9^, ^10^

The purpose of our prospective study was to determine the percentage of CRC patients with low GTA-446 levels diagnosed by colonoscopy relative to the general population, as well as the relative risk of CRC associated with a low GTA-446 level.

**What's new?**

This study showed that 86% of CRC patients had low GTA-446 levels. The relative risk of CRC was higher in subjects with low than in those with normal GTA-446 levels. The lack of increased CRC incidence with age among patients with normal GTA-446 levels suggested that low GTA-446 levels could be driving the age increase in CRC incidence rates. GTA-446 testing should be considered as a novel CRC risk-stratification tool.

## Material and Methods

### Study protocol

The rationale for the study was to compare the CRC incidence rates of low *versus* normal serum GTA levels as a predictor of new CRC diagnoses among a random sample of subjects scheduled for colonoscopy for any reason. Between June 2008 and August 2010, 4,994 subjects undergoing colonoscopy at the Regina General and Pasqua Hospitals in Regina, Saskatchewan, were enrolled on a first-come first-serve basis as part of a prospective population-based study approved by the University of Saskatchewan and Regina Qu'Appelle Health Region ethics boards. All subjects provided written informed consent, and eligibility for the colonoscopy arm was defined as any person aged 18–80 scheduled for colonoscopy for any reason. Trained study nurses recorded patient demographics, medical histories and colonoscopy findings from source documentation and patient interviews, into study-specific case report forms in a consistent manner. Colonoscopies were performed by staff gastroenterologists and surgeons at the endoscopy suites of both hospitals. Colonoscopy reports were collected on all subjects as source documentation for GI findings. Subjects with blockages or incomplete procedures were excluded. The reference population comprised 964 randomly selected serum samples (no exclusion criteria) from Saskatchewan residents provided by the Saskatchewan Disease Control Lab collected using protocols consistent with those used for subjects undergoing colonoscopy. Subjects were considered to have positive GI symptomology if they had a condition falling into any of the following categories listed in either the pre- or postoperative reports, pathology report, medical history source documents, recall and/or symptoms presenting at the time of the procedure: confirmed CRC diagnosis, any GI acute or chronic inflammatory condition, inflammatory bowel disease (IBD) (Crohn's, ulcerative colitis), diverticulitis, the presence of polyps, GI bleeding, overt or occult blood in stool, ulcers, GI pain, follow-up owing to a GI lesion or mass or abnormality, physical abnormality, previous GI cancer, previous GI surgery, upper GI abnormality and/or abnormal GI lab or genetic marker (such as hereditary non-polyposis colorectal cancer, *etc*.).

### Sample analysis

All serum samples were treated and processed equally. Samples were extracted as described previously with slight modification.^9^ Briefly, 0.31 μg/mL ^13^C cholic acid in 1% NH_4_OH was added to 160 μL of serum followed by two sequential extractions with 1 mL ethyl acetate. The extract was acidified with 4% formic acid followed by two more ethyl acetate extractions. Following centrifugation after each extraction, the total ethyl acetate pool was diluted 1:5 with water-saturated ethyl acetate and analyzed by flow-injection tandem mass spectrometry using an Applied Biosystems Q-Trap 4000 as described previously.^9^ Raw data were acquired using Analyst 1.5, converted to ASCII format and peak intensity determined as the mean of the intensity of the 92nd to 98th percentile of points comprising the ion chromatogram. The concentration of GTA-446, expressed as ^13^C cholic acid equivalents, was calculated by extrapolation from a ^13^C cholic acid standard curve. Precision was deemed acceptable if four control samples spaced equally among every 96 patient samples showed a percent CV of <15%, and if the *R*^2^ of the standard curve was >0.98. Accuracy was deemed acceptable if at least four out of the six standard curve points were between 80 and 120% accurate.

### Statistical analysis

The sample size of the trial was powered on the *a priori* expectation that the GTA-446 test would achieve a sensitivity of 75% at a specificity of 90%,^8^ and that the historical CRC yield of colonoscopy in Saskatchewan was approximately 2%. Results of comparisons (based on two-tailed Student's *t*-test) and regression analysis were performed with Microsoft Excel and considered significant if the *p*-value was <0.05 and the *F*-stat was >4. Data summarized in Table 3 were used for the determination of risk ratios and χ-squared *p*-values. Calculations were performed using SAS version 9.2 and JMP version 8.01.

## Results

### Patient characteristics

The baseline characteristics of the study population are summarized in Table 1. The study included a random sample of 964 age-distributed, geographically matched provincial serum samples from the Saskatchewan Disease Control Lab (the provincial clinical reference lab), and 4,994 samples from subjects undergoing colonoscopy on a first-come, first-serve basis between June 2008 and August of 2010. Eligible subjects had to be between 18 and 80 years of age, scheduled for a colonoscopy and able to provide written consent. There was no exclusion criteria based on prior cancer history or GI indication, resulting (as expected) in a population prevalent with pre-existing GI conditions and elevated CRC incidence. Of the 4,994 cases, 71 were excluded owing to incomplete collection of data, duplicate enrolment or incomplete procedures, resulting in a final study group of 4,923 subjects. Of these, 4,261 (86.6%) were positive for at least one overt GI symptom or condition (such as polyps, IBD, pain, blood in stool, inflammatory condition or other—**Methods**). Clinical data were recorded into case report forms based on the patient's medical records, patient recall or presentation of symptoms at the time of enrolment.

**Table 1:** Trial population baseline characteristics

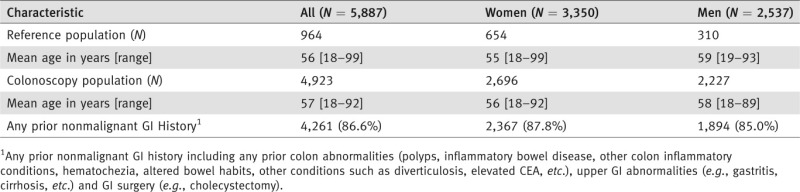

### Colonoscopy findings

The results of colonoscopy are summarized in Table 2. After colonoscopy, 3,388 (68.8%) of subjects were diagnosed with a GI-related condition, and 98 confirmed new cases of CRC were detected. The TNM stage distribution of the new cases was 30 stage 0/I, 22 stage II, 34 stage III and 12 stage IV. Among enrolled subjects, 1,575 (32.0%) were positive for at least one polyp, of which 30.6% were non-neoplastic, 41.0% had tubular adenomas, 7.5% had tubulovillous adenomas, 1.1% had villous adenomas and 5.3% had adenocarcinoma. Among subjects with polyps, 6.9% showed moderate- to high-grade dysplasia, whereas 84.4% had low-grade dysplasia. Among all subjects, 2.9% were diagnosed with IBD.

**Table 2:** Colonoscopy results

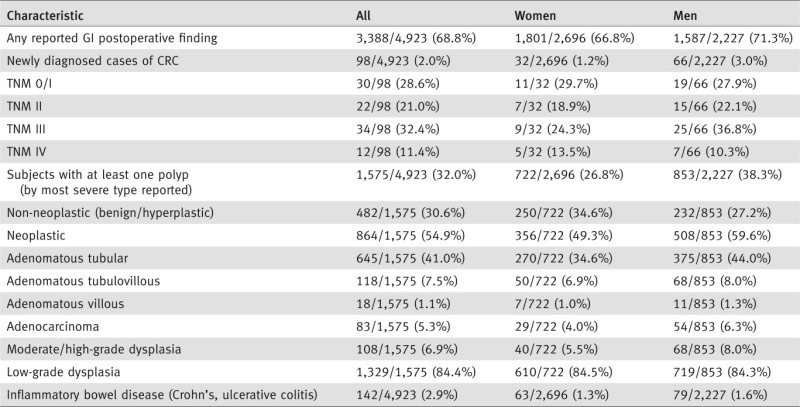

### Low GTA-446 positivity rates

Age is currently the most significant risk factor for CRC. Accordingly, a low-serum GTA-446 level was defined by the range encompassing the bottom 10th percentile of reference subjects with low-age-associated risk (those aged 40–49), which was below 0.35 μg/mL. The percent of subjects with low GTA-446 levels increased with age in both the reference and the colonoscopy populations; however, a higher percentage of subjects with low levels was observed across all ages for those undergoing colonoscopy compared to the reference population (Table 3).

**Table 3:** Percentages of subjects with low serum GTA-446 levels by age

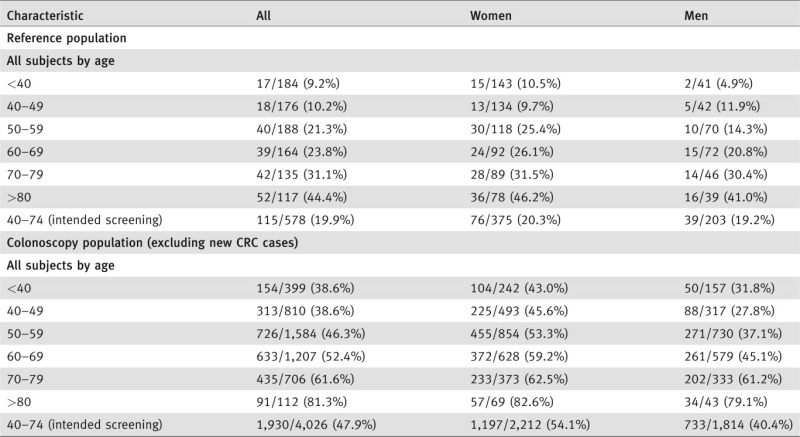

Of all subjects, those diagnosed with CRC showed the highest percentage of low GTA-446 levels (<0.35 μg/mL). Of 98 newly diagnosed cases, 84 (85.7%) showed low GTA-446 levels (Table 4). By stage, 23 out of 30 (76.7%) stage 0/I, 22 out of 22 (100%) stage II, 30 out of 34 (88.2%) stage III and 9 out of 12 (75%) stage IV subjects showed low-serum GTA-446 levels. When grouped as either early-stage (0–II) or late-stage (III–V), 86.5% of early-stage patients and 84.8% of late-stage patients showed low-serum GTA-446 levels. There was no significant difference between the percentage of low GTA-446 levels for early- *versus* late-stage disease (*p* > 0.05).

**Table 4:** Percentages of subjects with low-serum GTA-446 levels by pathological finding

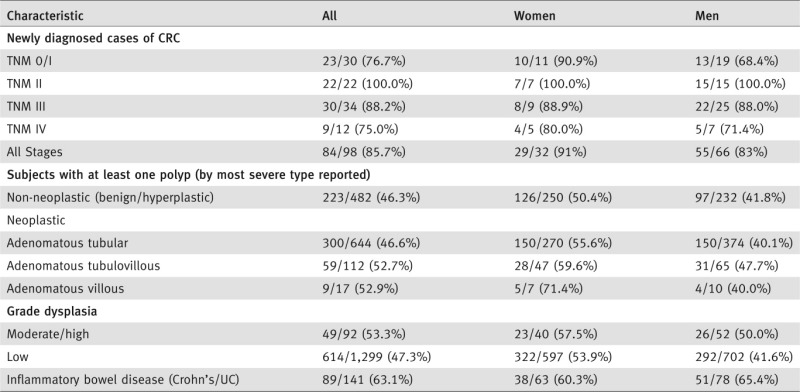

In subjects with at least one reported polyp, the low GTA-446 positivity rate was 46% for those with hyperplastic polyps and 53% for those with tubulovillous and villous adenomas (Table 4). The low GTA-446 positivity rate for subjects with low-grade dysplasia was 47% and 53% for those with moderate- to high-grade dysplasia. The percentage of subjects with IBD and low GTA-446 levels was 63%, the next highest after diagnosed CRC.

### Relative risk and CRC incidence rates among subjects with low *versus* normal GTA-446 levels

**Figure 1:**
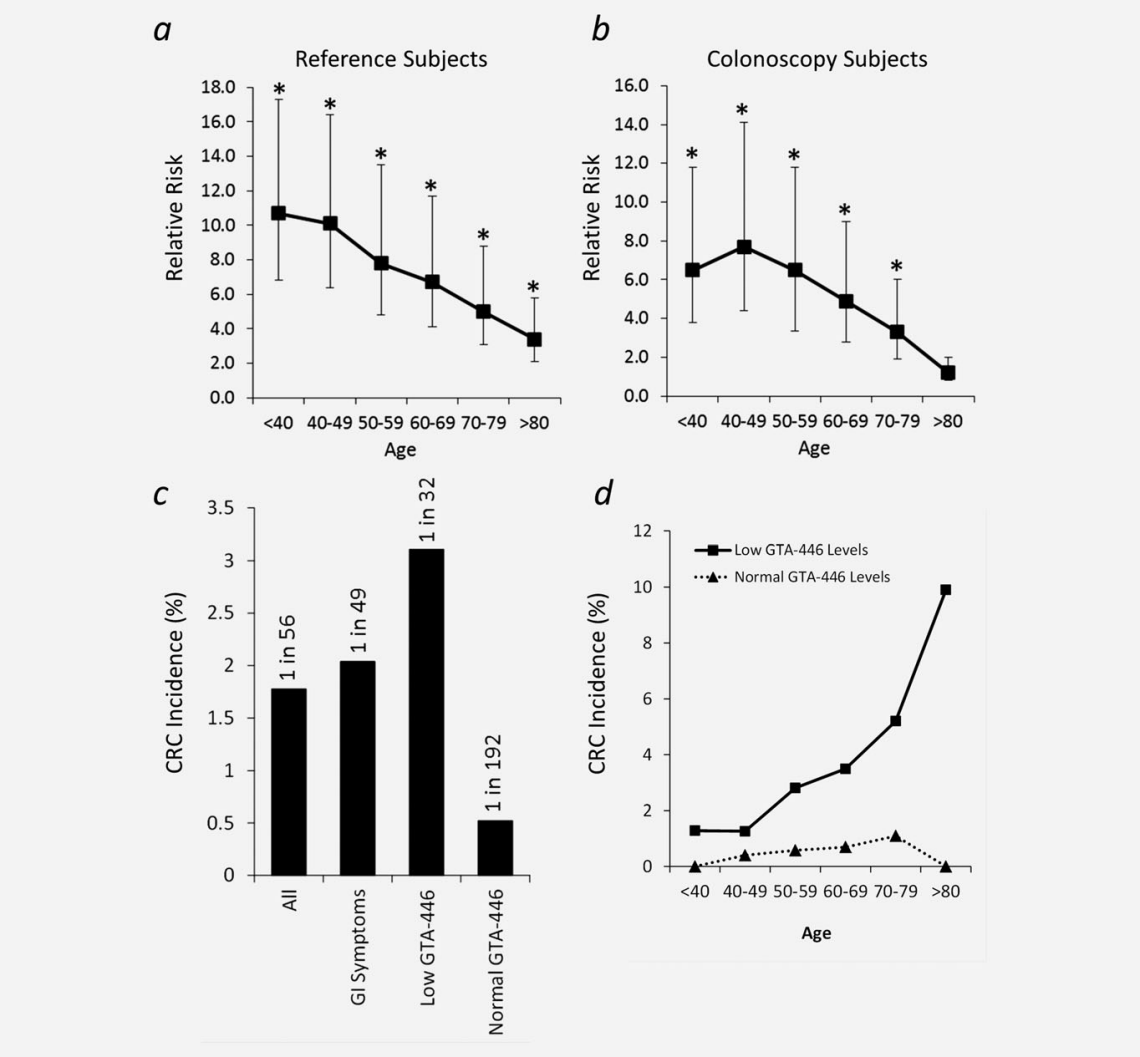
Relative risk and CRC incidences by age. Relative risk, based on the proportions of CRC and control cases with low *versus* normal GTA-446 levels (**Methods**), is shown by decade of life for the reference population (*a*) and for the colonoscopy population (*b*). Error bars represent the 95% C.I. and asterisks denote χ-squared *p*-values <0.0001. (*c*) Bar graph of CRC incidence rates for all subjects aged 40–74 who underwent colonoscopy, subjects with overt GI symptoms and subjects with low and normal GTA-446 levels. (*d*) Line plot of CRC incidence by decade of life for subjects undergoing colonoscopy based on GTA-446 level. For description, see **Results** section.

The relative risk of being diagnosed with CRC given a low GTA-446 level was determined by decade of life and was found to be inversely associated with age in both the reference and the colonoscopy populations (Figs. 1*a* and 1*b*, respectively). The relative risk of a person in the reference population aged 40–49 with a low GTA-446 level was 10.1 (95% C.I.: 6.4–16.4, *p* < 0.0001), which dropped by decade of life to 3.4 (95% C.I.: 2.1–5.8, *p* < 0.0001) by age 80 (Fig. 1*a*). Subjects undergoing colonoscopy had a similar risk profile, beginning with a relative risk of 7.7 (95% C.I.: 4.4–14.1, *p* < 0.0001) for subjects aged 40–49, and declining thereafter with age to a nonsignificant level of 1.2 (95% C.I.: 0.80–2.0, *p* = 0.46) by age 80 (Fig. 1*b*).

The CRC incidence rate among subjects with reported GI symptoms was compared to the incidence rate in subjects aged 40–74 with low and normal serum GTA-446 levels at an observational level. The CRC incidence rate for all subjects aged 40–74 in the study was approximately 1.75% (1 in 57). The incidence rate for subjects with GI symptoms was 2% (1 in 49), whereas the rate for subjects with low GTA-446 levels was just over 3% (1 in 32). The CRC incidence rate dropped to 0.5% (1 in 192) for symptomatic subjects with normal GTA-446 levels. This represented a 1.8-fold increase in CRC incidence in subjects with low GTA-446 levels compared to subjects with GI symptoms, a sixfold reduction in CRC incidence in subjects with normal GTA-446 levels *versus* subjects with low levels, and approximately a fourfold reduction in incidence for subjects with GI symptoms but normal GTA-446 levels (2 *versus* 0.5%, Fig. 1*c*).

The above analysis was then expanded by decade of life among subjects undergoing colonoscopy with low *versus* normal serum GTA-446 levels (Fig. 1*d*). As expected, the CRC incidence rate in subjects with low GTA-446 levels increased with age (*R* = 0.94, *F*-stat = 22, *p* = 0.019). However, there was no significant correlation between CRC incidence rate and age in subjects with normal GTA-446 levels (*R* = 0.11, *F*-stat = 0, *p* = 0.87). In other words, increasing age was not observed to be a significant CRC risk factor in subjects with normal GTA-446 levels. The CRC incidence rate in subjects aged 70–79 with normal GTA-446 levels was actually lower than the CRC incidence rate of subjects below age 50 with low GTA-446 levels (Fig. 1*d*). Therefore, the increased CRC incidence rate with age was highly associated with the increase in low serum GTA-446 levels with age.

## Discussion

The goal of a CRC screening program is to reduce mortality. Achieving this goal requires improvements in early-stage detection, where survival can be maximized by treatment. Current population-wide CRC screening programs are based on risk detection, which may be pathology based (such as blood in the stool, the presence of tumor markers, history of adenomas, abdominal pain, *etc*.), or nonpathology based (such as age or family history). However, age is the largest risk factor for CRC. By age 50, risk has accumulated to a magnitude (as measured by incidence) that warrants endoscopic examination for CRC presence independent of other risk factors. Considering that, in Canada, age-based compliance with screening colonoscopy guidelines is <20%, the vast majority of CRC cases are diagnosed only after symptoms occur that are severe enough to overcome a patient's aversion to colonoscopy.^11^–^13^ The early-stage detection rate for colon cancer in Canada is consequently only about 9% for stage I and 35% for stage II.^2^ Improving the detection rate of early-stage CRC through screening will therefore require tests that have early-stage sensitivity *and* public acceptance. It is worthwhile to point out that the voluntary enrolment compliance for our study, based on a simple blood test, was >95%.

In colonoscopy studies strictly controlled for asymptomatic subjects, early-stage detection rates of 59,^14^ 63^15^ and 73%^16^ have been reported, indicating that early-stage CRC is predominately asymptomatic. Unfortunately, 80% of asymptomatic subjects, for one reason or another, are not undergoing screening colonoscopy. The GTA-446 test offers several characteristics that could increase compliance in this population, including higher public acceptability over fecal tests or colonoscopy, high sensitivity for early-stage CRC and a plausible biological mechanism.

GTA-446 is a circulating bioactive long-chain fatty acid that represents a nonpathological selection criterion for identifying high-risk subjects who should subsequently undergo colonoscopy. Our study was performed to evaluate the CRC risk associated with low *versus* normal GTA-446 levels, from which several important findings emerged. First, 86% of newly detected CRC cases showed low-serum GTA-446 levels, independent of disease stage. This sensitivity was consistent with the results from our previous studies.^8^, ^9^ Over time, a test with early-stage sensitivity would result in a shift away from late-stage diagnoses, and consequently an increase in survival and a reduction in late-stage treatment costs.

Second, there was significantly elevated risk in subjects with low *versus* normal GTA-446 levels, which was inversely associated with age. The risk was the greatest for subjects under age 50 (relative risk of up to 10.1), suggesting that screening based on GTA-446 at an early age may be warranted. Although the risk declined consistently with age in both the colonoscopy and the reference populations as a function of the increased percentage of subjects with low GTA-446 levels with age, the risk was significant for all age groups examined in our study except for subjects having colonoscopy over age 80. For subjects already having colonoscopy, there was still an average sixfold difference in the CRC incidence rate between those with low *versus* normal GTA-446 levels <74 years of age. The fact that CRC risk was greater among subjects with low GTA-446 levels than those with overt GI symptomology puts into context this marker's potential utility for assessing risk across the general population, as well as for triaging endoscopy patients where resources may be limited.

Third, we speculated that that if the decline in GTA-446 levels with age contributed to the age-related increase in CRC risk, then the CRC incidence rate in subjects with normal GTA-446 levels should not correlate with age, which was indeed observed. This supports the premise that the increased CRC incidence rate with age may be owing to an increased incidence of low-serum GTA-446 levels with age. As far as we are aware, this is the first report of a biomarker that can stratify a population such that age is no longer a relevant risk factor for CRC. Put into context, a person over 70 years of age with a normal GTA-446 level showed a lower risk of CRC than a person under the age of 50 with a low GTA-446 level.

The results of our study, as well as other data collected to date, suggest that GTA-446 plays a role in protecting the body against cancer. First, the lack of association between GTA-446 and disease stage in several studies, including this one, indicates that disease presence is not responsible for the reduction.^8^, ^9^ This was reaffirmed in patients prior to and after surgical tumor removal, and in patients following chemo and radiation therapy, who exhibited no restoration in GTA-446 levels after treatment.^9^ Second, there was a significant correlation between the rate of GTA-446 reduction with age and the increase in CRC incidence rate with age in the general population.^9^ Third, GTAs, including GTA-446 enriched from human serum, showed anticancer biological properties.^10^ For example, GTA-enriched extracts were shown to inhibit human colon cancer cell line growth in a pro-apoptotic manner.^10^ Inhibition of the inducible nitric oxide synthase system was also observed at the transcript, protein and enzyme level, as was IκBα induction and NFκB protein inhibition.^10^ Further evidence of an anti-inflammatory role was observed when GTA-pretreated RAW264.7 cells exhibited reduced levels of several proinflammatory markers upon LPS-mediated stimulation including NOS2, TNF-α, COX2 and IL-1β at the enzyme, protein and transcript level.^10^ We propose here that a decline in GTA-446 level with age over an extended period of time may represent a previously unknown mechanism by which the body's innate ability to protect itself against an accumulating chronic inflammatory state becomes compromised, which may contribute to the underlying inflammation associated with CRC.^17^–^19^ Whether low GTA-446 levels are associated, through an inflammatory cascade, to the well-established hallmark sequence of sporadically acquired genetic abnormalities accompanying most tumors is a valid theory worthy of further pursuit.^20^

Although the role of specific GTAs in other cancers has not been thoroughly investigated, unpublished results by our group show that GTA-446 is not reduced in liver, prostate or breast cancers. However, reduced levels of specific longer-chain 36-carbon GTAs have been observed in the serum of pancreatic and ovarian cancer patients (unpublished results). Further studies investigating the role of GTAs in these and other indications are clearly warranted. The concept of restoring GTA-446 levels, in a preventive manner, is another exciting opportunity worth exploring.

The primary challenge we faced in our study was choosing the appropriate reference population such that the GTA-446 test positivity rates would be representative of a true screening scenario (*i.e.*, the rates that would be expected if the test was actually used to screen the population). This was a challenge because the majority of studies normally investigate pathology-based risk factors by relying on colonoscopy findings to define the low-risk baseline and the true positive populations (*i.e.*, the absence and presence of pathology, respectively). However, most “average-risk” colonoscopy trials, for reasons unknown, still report higher than expected CRC incidence rates despite aggressive exclusion criteria.^21^ We therefore felt no need to exclude subjects based on prior risk or symptomology to create a “control” population, particularly in light of the fact that GTA-446 is not a pathology-based marker. As a high percentage of symptomatic cases was anticipated, and there was no way to estimate how many average-risk age-recommended screening colonoscopies were going to be enrolled in the study, the control group had to be defined by the distribution of the risk factor marker in the intended screening population. The limitation, however, was that because the samples were anonymous, we had to assume the CRC incidence rate would have been representative of the current population incidence rate, and negligible at less than one out of the 964 subjects. Certainly, the data showed that colonoscopies performed in Saskatchewan were highly biased toward diagnostic procedures, and not average-risk screening.

In conclusion, the results reported here suggest that measuring serum GTA-446 levels is useful as a nonpathology-based risk selection criterion for subsequent endoscopic examination, similar to measuring glucose as a risk marker for diabetic symptomology. As the CRC incidence rate in subjects aged 40–49 with low-serum GTA-446 levels was similar to the total population incidence rate in subjects aged 50–59 (1.3 *versus* 1.6%), measuring serum GTA-446 for identifying high-risk subjects under age 50 may be warranted. This would offer new hope to the approximately 6% of CRC patients currently diagnosed under the age of 50.^1^ Finally, the results of our study seriously question the independence of age as a CRC risk factor, and raise the possibility that advancing age is but a surrogate marker for the increased incidence of low-serum GTA-446 levels with age. A definitive answer to this question awaits the execution of a suitably designed longitudinal trial.

## Abbreviations

CRC: colorectal cancer
GI: gastrointestinal
IBD: inflammatory bowel disease
